# Hypothermia in spinal cord injury

**DOI:** 10.1186/cc11270

**Published:** 2012-06-07

**Authors:** Guadalupe A Castillo-Abrego

**Affiliations:** 1 Neurocritical Care Unit, Caja de Seguro Social Hospital, Panamá City, Panamá; 2 Medical Staff Critical Care Unit, Punta Pacifica Hospital: John Hopkins Affiliated Hospital, San Fernando Hospital, Centro Médico Paitilla, Hospital Nacional, Panamá City, Panamá

## Historical considerations

In 1862 Edwin Smith, Egyptologist, acquired a papyrus that was thought to be between 2,500 and 3,000 years old. It was translated in 1930 by James Breasted, and was found to contain information on medical therapies performed on 48 patients, including six cases of cervical spine trauma. In the papyrus these injuries were described as 'dislocation of the vertebrae of the neck with unconscious arms and legs, where urine was drained and the phallus was erect, and it was determined that this cannot be treated' [1].

Other physicians like Hippocrates and Claudius Galen made historical reference to the management of spinal cord injury. Galen studied injuries in gladiators, and described 'that injury of the spinal cord caused paralysis and loss of sensation below the level of injury ... and determined that high spinal cord injuries are incompatible with life' [2,3].

Chaulic Guy (1300 to 1360) carried out innovations in the management of traumatic bone injuries by introducing boards and suspensions to treat these fractures; however, he gave a pessimistic view on spinal injuries [4,5].

Ambrose Pare (1510 to 1590) developed spine surgery, and along with Hildanus Fabricius (1560 to 1634) used traction techniques, reduction and utilization of wood frames to treat cervical spine fractures [6].

Louis Pasteur's (1822 to 1895) surgical innovations and discoveries and the advent of asepsis and antisepsis by Semmelweis (1816 to 1868) and others, and primarily the application of general anesthesia by Morton (1819 to 1868) and others gave a new impulse to this field. Together these developments helped reduce surgical mortality and infections [5,7].

In the nineteenth century, anatomy and physiology of the central nervous system were studied. The neuron doctrine was developed by Santiago Ramon y Cajal (1852 to 1934), who demonstrated the individuality of nerve cells and the connections that they have with each other [8,9].

Alfred R Allen between 1908 and 1911 developed a reproducible and quantifiable model that allowed induction of a uniform traumatic injury to the spine, and also explained the pathophysiological changes seen in spinal trauma, including key aspects of secondary spinal cord injury. In 1972 Tarlov showed that symptoms caused by compression of the spinal cord for more than 12 hours could sometimes still be reversed. During this period 'progressive central hemorrhagic necrosis' was identified, which consists of bleeding from the gray matter of the spinal cord central necrosis and destruction of white matter, with subsequent cavitations [10,11]. Finally, one of the possible immunomodalities of neuroprotection was suggested in 1968 by Albin and White who applied local hypothermia with favorable results in animals [12]. However, use of hypothermia was limited due to fear of side effects [13-17].

These preliminary studies were difficult to interpret due to the limited number of patients, lack of controls, concomitant surgical procedures and the concomitant use of drugs such as methylprednisolone [18]. In recent years there has been a renewed interest in the use of moderate therapeutic hypothermia due to its demonstrated neuroprotective effects in other areas. In Panama, we have been administering therapeutic hypothermia in selected cases with severe spinal cord injury. More frequently we apply fever management through endovascular, surface pads or medical thermomodulation protocols.

## Epidemiological aspects of spinal cord injury

No accurate numbers on spinal cord injury are available for Panama and Central America. In the United States there are around 12,000 new cases of traumatic spinal cord injury each year, not including those who die at the scene of the accident [19]. Approximately 1.3 million Americans have some type of chronic paralysis resulting from spinal cord injury [20,21]. The leading causes are motor vehicle accidents (41.3%), falls (27.3%), acts of violence (15%) and recreational activities (7.9%). Apart from the medical and personal consequences for the patient, the economic impact in terms of ability to work is significant.

### Spinal cord injury, neurophysiological aspects - how can therapeutic hypothermia help?

We will provide a concise description of the pathophysiological changes that occur after a spinal injury that might justify use of therapeutic hypothermia to mitigate ongoing destructive processes.

Spinal cord injury is a process that can be divided into three phases [22,23]. The *primary mechanism *refers to the direct mechanical damage. This may include compression, stretching, persistent concussion, contusion, compression and laceration of the spinal cord [22,23]. The *secondary mechanism *involves a cascade of events at the cellular level triggered by the primary mechanism. These biochemical events are responsible for increased tissue damage and promote the apoptotic cascade. These include those listed in Table 1. The *healing mechanism *(third mechanism) begins in the days after injury and can last for months or years. This can paradoxically increase the neurological damage. Healing in the primary injury takes place by neutrophils, macrophages and lymphocytes, reactive astrocytes, Schwann cells, meningeal fibroblasts, and microglia invasion. Scar formation forms a barrier for cellular and molecular axonal regeneration.

**Table 1:** 

1. After the death of an oligodendrocyte, a progressive Walerian degeneration takes place from the site of primary injury, compromising the integrity of axons and mitochondria. Simultaneously caspase activation and apoptosis occur, leading finally to demyelination and release of nerve growth factor.
2. Vascular ischemia, impaired autoregulation, neurogenic shock, radial and axial hemorrhage, compromised microcirculation, collapsed veins, venous obstruction, vasospasm and thrombosis.
3. Ionic alterations: intracellular and extracellular calcium overload, increase of sodium and potassium.
4. Accumulation of neurotransmitters such as dopamine, norepinephrine, serotonin and glutamate at the extracellular level.
5. Release of arachidonic acid, production of free radicals and lipid eicosanoid peroxidation.
6. Endogenous opioid activation
7. Formation of cytotoxic and vasogenic edema
8. (Hyper)inflammatory response
9. Failure of ATP-dependent intracellular processes

Therapeutic hypothermia has been used in various types of neurological injury such as stroke, post-anoxic encephalopathy, and traumatic brain injury. Some of these results have been extrapolated to spinal cord trauma patients.

Moderate therapeutic hypothermia has been shown to affect the apoptotic cascade as well as other destructive mechanisms, ranging from improved energy balance, reduction of mitochondrial dysfunction, decreased vascular permeability and capillary leakage, mitigation of cell membrane injury, improvement of intracellular acidosis, mitigation of DNA injury, reduction in metabolic demand, and a decrease in proinflammatory cytokine and free radical release [24-28].

It is important to realize that there is a window time, perhaps several hours to days after the injury, during which this treatment modality can influence the course of events [29].

## Experimental studies and supporting evidence

Yu and colleagues used therapeutic hypothermia (33°C) after 30 minutes of ischemic injury to the thoracic spinal cord in rats, and reported improvement of motor function at the microscopic level associated with cooling [30]. Others reported improvements in functional outcome in various animal models associated with cooling [30-32]. In an animal study of spinal cord ischemia, hypothermia increased the duration of ischemia required to produce neurological deficits [31].

Moderate hypothermia has also shown potential benefits in invasive procedures including aortic clamp cross-clamping during thoracic surgery [33,34]. This can be regarded as indirect evidence that hypothermia could protect the spinal cord from ischemic injury, although the mechanisms in traumatic injury may be different from surgical trauma and ischemia.

Fever is a common complication in patients with spinal cord injury [35-37]. Of the identifiable etiologies the most common cause is infection (especially pneumonia and urinary tract infections). However, fever of unknown origin is the most frequent diagnosis, occurring in 66% of patients. It should be realized that patients with spinal cord injury have a high incidence of thermoregulatory problems, which can contribute to the high incidence of FUO [38,39].

Of note, Yu and colleagues demonstrated in an experimental study in rats that post-traumatic hyperthermia in thoracic spinal cord injury worsened behavioral and histopathological damage compared with normothermia, and was associated with an increase of overall contusion volume by increasing the vulnerability of both gray and white matter structures compared with normothermia [40].

### Role of moderate hypothermia in the clinical scenario of patients with spinal cord injury

Early clinical studies with irrigation to induce local hypothermia produced conflicting results [41]. However, the advent of more reliable cooling devices that can better maintain body temperature within a predetermined range have improved our ability to deliver targeted therapy.

Hypothermia has been used to protect the spinal cord and prevent paraplegia during high aortic cross surgery. In one study with long-term follow-up, the incidence of spinal cord injury in patients undergoing high-risk thoracoabdominal aneurysm repair under hypothermia over a 10-year period the risk of SCI was 18% in hypothermic patients compared with 29% in historical controls (*P *<0.01) [29,33].

Interest in hypothermia for spinal cord injury received a boost when in 2007 a high-profile case was reported. An NFL player suffered a complete AIS A cervical spine injury, and was treated immediately on the football field with moderate hypothermia. This individual had a much better than expected outcome and this led to a spate of publications on the potential use of hypothermia for spinal cord injury [40,42-44]. Of course, such anecdotal evidence cannot prove the benefit of any therapy, and professional organizations such as the American Association of neurological surgeons, Neurological and Spine Surgery Joint Sections and Joint Section of Trauma concluded that there was currently not enough evidence available to recommend or discourage the practice of therapeutic hypothermia as a treatment for spinal cord injury [42,45-47].

In 2009 Levi and colleagues successfully tested the safety and feasibility of systemic hypothermia induction in spinal cord injury with an endovascular cooling device [48]. The authors treated 14 patients with AIS A spinal cord injury with moderate hypothermia. At a median follow-up of 1 year they found an improved conversion rate: in 6/14 cooled patients there was an improvement of the neurological examination (three patients improved to AIS B, two patients improved to AIS C and one patient improved to AIS D). This represents an improvement rate of 42.8%, higher than the 12.5 to 20% found in various other studies where patients had not been treated with hypothermia [49,50]. Complications associated with cooling were mostly respiratory issues (atelectasis and pneumonia) but these rates were similar in the other studies where cooling had not been used. Adverse events such as coagulopathy, deep venous thrombosis and pulmonary embolism were not reported in the patients treated with hypothermia. This is the first investigation on the safety systemic cooling in acute spinal cord injury [48,51].

To determine efficacy will require randomized controlled clinical studies. Currently there are plans to organize such a trial, which will involve 17 centers to determine whether moderate hypothermia improves outcome in a larger population of patients with acute spinal cord injury. Details can be found at online: http://www.miamiproject.org. The protocol calls for induction of hypothermia within 6 hours of injury, to be maintained by endovascular cooling, where they will evaluate the safety of different durations of hypothermia, outcomes and risks [47].

Fever in patients with spinal cord injury, if not controlled promptly, may lead to increased morbidity and mortality because of hyperthermic damage to cells. Therefore controlling fever is an important goal of care in these patients. Preliminary data suggest that endovascular cooling can be used effectively for this purpose [52,53].

There are no proven treatments with high grades of scientific evidence for the devastating consequences of spinal cord injury. In Panama, selected patients with ASIA A lesions are treated with therapeutic cooling for a period of 24 to 48 hours. This can be done in either mechanically ventilated or nonventilated patients. Other aspects of treatment include keeping adequate medullar perfusion pressure, normothermia throughout their ICU stay (accomplished by pharmacological interventions, mechanical cooling either with a surface cooling or endovascular device), early enteral immunonutrition, and various tests such as somatosensory evoked potentials.

## Conclusion

So far there are no proven therapeutic interventions that improve outcome in severe spinal cord injury. Hypothermia appears to be a promising treatment in this population, and needs to be studied in prospective clinical trials. Fever control should be a goal of care in these patients.

## References

[B1] Wilkins RH Neurosurgical classic XVII. Edwin Smith surgical papyrus J Neurosurg 1964 24 240 244 14127631 10.3171/jns.1964.21.3.0240

[B2] Marketos SG Skiadas PK Hippocrates. The father of spine surgery Spine 1999 24 1381 1387 10.1097/00007632-199907010-00018 10404583

[B3] Marketos SG Skiadas PK Galen: a pioneer of spine research Spine 1999 24 2358 2362 10.1097/00007632-199911150-00012 10586461

[B4] Goodrich JT History of spine surgery in the ancient and medieval worlds Neurosurg Focus 2004 16 1 3 15264780 10.3171/foc.2004.16.1.3

[B5] Lifshutz J Colohan A A brief history of therapy for traumatic spinal cord injury Neurosurg Focus 2004 16 1 8 15264783

[B6] Sontang VKH Greenblatt SH History of degenerative and traumatic disease of the spine A History of Neurosurgery in its Scientific and Professional Contexts 1997 Parke Ridge, IL: American Association of Neurological Surgeons 355 371

[B7] Knoeller SM Seifried C Historical perspective. History of spinal surgery Spine 2000 25 2838 2843 10.1097/00007632-200011010-00020 11064533

[B8] McHenry LC Garrison's History of Neurology 1960 Springfield, IL: Charles C. Thomas 265 424

[B9] Naderi S Ture U Pait TG History of spinal cord localization Neurosurg Focus 2004 16 1 6 15264793 10.3171/foc.2004.16.1.16

[B10] Collins WF Narayan RK, Wilberger JE, Povlishock JT Historical perspective on spinal cord injury Neurotrauma 1995 New York: McGraw Hill 1041 1047

[B11] Tarlov I Acute spinal cord compression paralysis J Neurosurg 1972 36 10 20 10.3171/jns.1972.36.1.0010 5007267

[B12] Albin MD White RJ Acosta Rua G Study of functional recovery produced by delayed localized cooling after spinal cord injury in primates J Neurosurg 1968 29 113 120 10.3171/jns.1968.29.2.0113 4970575

[B13] Bricolo A Ore GD Da Pian R Local cooling in spinal cord injury Surg Neurol 1976 6 01 106 951644

[B14] Demian YK White RJ Yashon D Anesthesia for laminectory and localized cord cooling in acute cervical spine injury. Report of three cases Br J Anaesth 1971 43 973 979 10.1093/bja/43.10.973 4940055

[B15] Koons D Gildenberg PL Dohn DF Local hypothermia in the treatment of spinal cord injuries. Report of seven cases Clev Clin Q 1972 39 109 117 10.3949/ccjm.39.3.109 5084376

[B16] Selker RG Icewater irrigation of the spinal cord CH Surg Forum 1971 22 411 413 5121424

[B17] Tator CH Deecke L Value of normothermic perfusion, hypothermic perfusion and durotomy in the treatment of experimental acute spinal cord trauma J Neurosurg 1973 39 52 64 10.3171/jns.1973.39.1.0052 4197833

[B18] Hansebout RR Kuchner EF Effect of local hypothermia and of steroids upon recovery from experimental spinal cord injuries compression injury Surg Neurol 1975 4 531 536 1188593

[B19] Spinal cord injury, facts and figures at a glance J Spinal Cord Med 2008 31 357 358 18795487

[B20] Paralysis and Spinal Cord Injury in the United States 2009 Short Hills, NJ: Christopher and Dana Reeve Foundation

[B21] Spinal cord injury facts and figures at a glance J Spinal Cord Med 2010 33 439 440 21061906

[B22] Hagg T Oudega M Degenerative and spontaneous regenerative process after spinal cord injury J Neurotrauma 2006 23 248 261 10.1089/neu.2006.23.263 16629615

[B23] Mc Donald J Belegu V De myelization and remyelination after spinal cord injury J Neurotrauma 2006 23 345 359 10.1089/neu.2006.23.345 16629621

[B24] Polderman KH Therapeutic hypothermia in the intensive care unit: problems, pitfalls and opportunities (review). Part 1: indications and evidence Intensive Care Med 2004 30 556 575 10.1007/s00134-003-2152-x 14767591

[B25] Mayer SA Sessler DI Therapeutic Hypothermia 2005 1 New York: Marcel Dekker

[B26] Hayashi N Dietrich DW Brain Hypothermia Treatment 2004 1 Tokyo: Springer-Verlag

[B27] Polderman KH Paradis N, Halperin H, Kern K, Wenzel V, Chamberlain D Mechanism and potential side effects of therapeutic mild hypothermia Cardiac Arrest: The Science and Practice of Resuscitation Medicine 2007 2 Cambridge: Cambridge University Press 859 870

[B28] Edwards P Arango M Balica L CRASH trial collaborators. Final results of MRC CRASH, a randomized placebo controlled trial of intravenous corticosteroid in adult with head injury - outcomes at 6 months Lancet 2005 365 1957 1959 15936423 10.1016/S0140-6736(05)66552-X

[B29] Polderman KH Induced hypothermia and fever control for prevention and treatment of neurological injuries Lancet 2008 371 1955 1969 10.1016/S0140-6736(08)60837-5 18539227

[B30] Yu GC Jimenez O Marcillo AE Beneficial effects of modest systemic hypothermia on locomotors function and histopathological damage following contusion-induced spinal cord injury in rats J Neurosurg 2000 93 85 93 10.3171/spi.2000.93.1.0085 10879763

[B31] Pearse DD Lo TP Jr Cho KS Histopathological and behavioral characterization of a novel cervical spinal cord displacement contusion injury in the rat J Neurotrauma 2005 22 680 702 10.1089/neu.2005.22.680 15941377

[B32] Lo TP Cho KS Grag MS Systemic hypothermia improves histological and functional outcome after cervical spinal cord contusion in rats J Comp Neurol 2009 514 433 448 10.1002/cne.22014 19350644

[B33] Hartemink K Wisselink W Rauwerda J Girbes A Polderman K Novel applications of therapeutic hypothermia: report of three cases Crit Care 2004 8 R343 R346 10.1186/cc2928 15469578 PMC1065027

[B34] Fehrenbacher JW Hart DW Huddleston E Optimal end organ protection for thoracic and thoracoabdominal aortic aneurysm repair using deep hypothermia circulatory arrest Ann Thorac Surg 2007 83 1041 1046 10.1016/j.athoracsur.2006.09.088 17307456

[B35] Mc Kinley W McNamee S Meade M Kandra K Incidence, etiology and risk factors for fever following acute spinal cord injury J Spinal Cord Med 2006 29 501 506 17274488 10.1080/10790268.2006.11753899 PMC1949035

[B36] Ulger F Dilek A Karakaya D Senel A Fatal fever of unknown origin in acute cervical spinal cord injury: five cases J Spinal Cord Med 2009 32 343 348 19810636 10.1080/10790268.2009.11760788 PMC2718819

[B37] Scott SR Matthew MJ Philip MS William LB Fatal malignant hyperpyrexia in a cervical spine-injured patient J Trauma 2005 58 375 377 10.1097/01.TA.0000066349.88810.97 15706204

[B38] Montgomerie JZ Infections in patients with spinal cord injuries Clin Infect Dis 1997 25 1285 1290 10.1086/516144 9431366

[B39] Sugarman B Brown D Musher D Fever and infection in spinal cord injury patients JAMA 1982 248 66 70 10.1001/jama.1982.03330010040028 7087094

[B40] Yu CG Jagid J Ruenes G Detrimental effects of systemic hyperthermia on locomotor function and histophatological outcome after traumatic spinal cord injury in the rat Neurosurgery 2001 49 152 159 11440437 10.1097/00006123-200107000-00023

[B41] Kwon BK Mann C Sohn HM hypothermia for spinal cord injury Spine 2008 371 1955 1969 10.1016/j.spinee.2007.12.006 18329959

[B42] Dietrich WD Presidential address Presented at 34th Annual Meeting of the Cervical Spine Research Society; 30 November 2006; Florida, USA

[B43] Goldstein J Lower Inf Body Temperature Shows Promise for Trauma Treatment http://sci-info-pages.com/2006/05/lowering-body-temp-shows-promise-for.html

[B44] Hartemink KJ Wisselink W Rauweda JA Novel applications of therapeutic hypothermia: report of three cases Crit Care 2004 8 R343 R346 10.1186/cc2928 15469578 PMC1065027

[B45] Cappuccino A Bisson LJ Carpenter B The use of systemic hypothermia for the treatment of an acute cervical spinal cord injury in a professional football player Spine 2010 35 E57 E62 10.1097/BRS.0b013e3181b9dc28 20081503

[B46] Dietrich DW Therapeutic hypothermia for spinal cord injury Crit Care Med 2009 12 183 187 10.1097/CCM.0b013e3181aa5d85 19535953

[B47] Dietrich D Cappucino A Capuccino H Systemic hypothermia for the treatment of acute cervical spinal cord injury in sports Curr Sports Med Rep 2011 10 50 54 21228652 10.1249/JSR.0b013e318205e0b3

[B48] Levi A Barth Green Wang M Clinical application of modest hypothermia after spinal cord injury J Neurotrauma 2009 26 407 415 10.1089/neu.2008.0745 19271964

[B49] Geisler FH Coleman WP Crieco G Poonian D Sygen Study Group The Sygen multicenter acute spinal cord injury study Spine 2001 26 24 Suppl S87 S98 11805614 10.1097/00007632-200112151-00015

[B50] Van Middendorp JJ Hosman AJ Pouw MH EM-SCI Study Group ASIA impairment scale conversion in traumatic SCI: is it related with the ability to walk? A descriptive comparison with functional ambulation outcome measures in 273 patients Spinal Cord 2009 47 555 560 10.1038/sc.2008.162 19104512

[B51] Levi A Casella G Clinical outcome using modest intravascular hypothermia after acute cervical spinal cord injury Neurosurgery 2010 66 670 677 10.1227/01.NEU.0000367557.77973.5F 20190669

[B52] Marion DW Controlled normothermia in neurologic intensive care Crit Care Med 2004 32 S43 S51 10.1097/01.CCM.0000110731.69637.16 15043227

[B53] Tripathy W Whitehead C Endovascular cooling for severe hyperthermia in cervical spine injury Neurocrit Care 2011 15 525 528 10.1007/s12028-011-9529-4 21442387

